# Supplementary material for: From sequence to enzyme mechanism using multi-label machine learning
Source: BMC Bioinformatics. 2014 May 19;15:150. doi: 10.1186/1471-2105-15-150 (PMC4229970; doi:10.1186/1471-2105-15-150)
Supplement: Additional file 2 — Java code of ml2db. Additional file ml2db_code.tar.gz contains the Java source code to run the multi-label machine learning experiments and save the results to database. The code’s Javadoc is included. [file 1471-2105-15-150-S2.zip › additional file 2/ml2db/ml2db/doc/main/ml2db/class-use/DbManagers.html]

Uses of Class main.ml2db.DbManagers


JavaScript is disabled on your browser.


- Overview
- Package
- Class
- Use
- Tree
- Deprecated
- Index
- Help

- Prev
- Next

- Frames
- No Frames

- All Classes

## Uses of Class main.ml2db.DbManagers

- Packages that use DbManagers

  | Package | Description |
  | --- | --- |
  |  |  |
  | --- | --- |
  | main.ml2db |  |
  | main.ml2db.al.learning |  |
  | main.ml2db.mulan.attributesfiltered |  |
  | main.ml2db.mulan.learn |  |
  | main.ml2db.mulan.learn.crosseval |  |
  | main.ml2db.mulan.learn.traintest |  |
  | main.ml2db.mulan.mulanarff |  |
  | main.ml2db.mulan.mulanarff.database |  |
  | main.ml2db.weka |  |
  | test.ml2db |  |
  | test.ml2db.alltests |  |
  | test.ml2db.dataharness |  |
  | test.ml2db.weka |  |
- - ### Uses of DbManagers in main.ml2db

    Methods in main.ml2db that return DbManagers

    | Modifier and Type | Method and Description |
    | --- | --- |
    |  |  |
    | --- | --- |
    | `static DbManagers` | ProjectParameters.`getLocalDbManagers()` |
    | `static DbManagers` | ProjectParameters.`getRemoteDbManagers()` |
  - ### Uses of DbManagers in main.ml2db.al.learning

    Methods in main.ml2db.al.learning that return DbManagers

    | Modifier and Type | Method and Description |
    | --- | --- |
    |  |  |
    | --- | --- |
    | `DbManagers` | ExperimentDetails.`getArffAndResultsDbManagers()` |

    Constructors in main.ml2db.al.learning with parameters of type DbManagers

    Constructor and Description

    `AlExperimenter(mulan.data.MultiLabelInstances trainSet,
    mulan.data.MultiLabelInstances testSet,
    DbManagers dbMan,
    MulanLearner learner,
    java.lang.String resultsDirectory,
    ActiveLearning experiment)`

    `ExperimentDetails(IdMultiLabelInstances unlabelled,
    MulanLearner algorithm,
    java.lang.String distanceFunction,
    DbManagers dbManagers,
    UtilityMetric utilityMetric,
    java.lang.String imagesPath)`

    With set of unlabelled instances (no arff record identifier)

    `ExperimentDetails(int labelledArffId,
    int unlabelledArffId,
    MulanLearner algorithm,
    java.lang.String distanceFunction,
    DbManagers dbManagers,
    UtilityMetric utilityMetric,
    java.lang.String imagesPath)`

    Both labelled and unlabelled arff record identifier.

    `ExperimentDetails(int unlabelledArffId,
    MulanLearner algorithm,
    java.lang.String distanceFunction,
    DbManagers dbManagers,
    UtilityMetric metric,
    java.lang.String imagesPath)`

    Only Unlabelled arff record identifier.
  - ### Uses of DbManagers in main.ml2db.mulan.attributesfiltered

    Methods in main.ml2db.mulan.attributesfiltered that return DbManagers

    | Modifier and Type | Method and Description |
    | --- | --- |
    |  |  |
    | --- | --- |
    | `DbManagers` | AttFilter.`getDbManagers()` |

    Methods in main.ml2db.mulan.attributesfiltered with parameters of type DbManagers

    | Modifier and Type | Method and Description |
    | --- | --- |
    |  |  |
    | --- | --- |
    | `static int[]` | AttFilter.`run(int oldTrainArffId, int oldTestArffId, DbManagers dbMan)` Filter the old test arff by the train arff given and regenerate the old train set using all train+test classes. |

    Constructors in main.ml2db.mulan.attributesfiltered with parameters of type DbManagers

    Constructor and Description

    `AttFiltArff(java.lang.String dataPropsFileName,
    DbManagers dbMan,
    AttFilter filterManager,
    int oldArffRecordId)`

    `AttFilter(int oldTrainArffId,
    int oldTestArffId,
    DbManagers dbMan)`
  - ### Uses of DbManagers in main.ml2db.mulan.learn

    Methods in main.ml2db.mulan.learn with parameters of type DbManagers

    | Modifier and Type | Method and Description |
    | --- | --- |
    |  |  |
    | --- | --- |
    | `static java.lang.String` | MulanSerializer.`run(int trainArffId, MulanLearner learner, DbManagers dbMan, java.lang.String folderForModel)` |
  - ### Uses of DbManagers in main.ml2db.mulan.learn.crosseval

    Methods in main.ml2db.mulan.learn.crosseval that return DbManagers

    | Modifier and Type | Method and Description |
    | --- | --- |
    |  |  |
    | --- | --- |
    | `DbManagers` | CrossInputManager.`getDbManagers()` |

    Methods in main.ml2db.mulan.learn.crosseval with parameters of type DbManagers

    | Modifier and Type | Method and Description |
    | --- | --- |
    |  |  |
    | --- | --- |
    | `void` | CrossExperimenter.`setInputManager(int trainArffRecordId, DbManagers dbManagers)` |

    Constructors in main.ml2db.mulan.learn.crosseval with parameters of type DbManagers

    Constructor and Description

    `CrossExperimenter(DbManagers dbMan,
    int trainArffRecordId,
    MulanLearner learner,
    java.lang.String resultsFolder)`

    `CrossExperimenter(IdMultiLabelInstances trainSet,
    MulanLearner learner,
    DbManagers dbMan,
    java.lang.String resultsFolder)`

    `CrossInputManager(int trainArffRecordId,
    DbManagers dbMan,
    CrossExperimenter crossExperimenter)`

    Gets the instances from database arff record.
  - ### Uses of DbManagers in main.ml2db.mulan.learn.traintest

    Methods in main.ml2db.mulan.learn.traintest with parameters of type DbManagers

    | Modifier and Type | Method and Description |
    | --- | --- |
    |  |  |
    | --- | --- |
    | `static void` | TrainTestFullRun.`fullRun(DbManagers dbs, boolean filtered, boolean serialise, boolean predict, boolean saveTrueNegatives, int train, int test, MulanLearner learner)` Executes: 1. |
    | `static void` | TrainTestExperimenterSerialized.`runWithSerializedTrainedModel(DbManagers arffStoreAndResultsDbManagers, int trainArffRecordId, int testArffRecordId, MulanLearner learner, java.lang.String trainedModelFile, java.lang.String resultsFilesDirectory, boolean predict, boolean saveTrueNegatives)` |
    | `void` | TrainTestExperimenter.`setInputManager(int trainArffRecordId, DbManagers dbMan)` |
    | `void` | TrainTestExperimenter.`setInputManager(int trainArffRecordId, DbManagers dbMan, TrainTestExperimenter trainTestExperimenter)` |
    | `void` | TrainTestInputManager.`setTestArffRecord(int testArffId, DbManagers dbMan)` |
    | `static java.lang.String` | TrainTestExperimenterSerialized.`trainAndSerialize(int trainArffRecordId, MulanLearner learner, DbManagers dbManagers, java.lang.String directoryForSerializedModel)` |

    Constructors in main.ml2db.mulan.learn.traintest with parameters of type DbManagers

    Constructor and Description

    `TrainTestExperimenter(DbManagers dbMan,
    int trainArffRecordId,
    int testArffRecordId,
    MulanLearner learner,
    java.lang.String resultsDirectory)`

    `TrainTestExperimenter(IdMultiLabelInstances trainSet,
    IdMultiLabelInstances testSet,
    DbManagers dbManagers,
    MulanLearner learner,
    java.lang.String resultsDirectory)`

    `TrainTestExperimenterSerialized(DbManagers dbMan,
    int trainArffRecordId,
    int testArffRecordId,
    MulanLearner learner,
    java.lang.String trainedModelFile,
    java.lang.String resultsDirectory)`

    `TrainTestInputManager(int trainArffRecordId,
    DbManagers dbMan,
    TrainTestExperimenter trainTestExperimenter)`

    `TrainTestInputManager(int trainArffRecordId,
    int testArffRecordId,
    DbManagers dbMan,
    TrainTestExperimenter experimenter)`
  - ### Uses of DbManagers in main.ml2db.mulan.mulanarff

    Methods in main.ml2db.mulan.mulanarff that return DbManagers

    | Modifier and Type | Method and Description |
    | --- | --- |
    |  |  |
    | --- | --- |
    | `DbManagers` | IdMultiLabelInstances.`getDbMan()` |

    Methods in main.ml2db.mulan.mulanarff with parameters of type DbManagers

    | Modifier and Type | Method and Description |
    | --- | --- |
    |  |  |
    | --- | --- |
    | `static int[]` | MulanArff.`run(java.lang.String[] arffPaths, DbManagers dbMan, boolean massive)` Generates a number of ARFF files (the database the records are written is the same for all ARFF files) |
    | `static int` | MulanArff.`run(java.lang.String arffPropsPath, DbManagers dbMan, boolean massive)` Generates the ARFF (and XML) files and returns the identifier of the arff record in the database |

    Constructors in main.ml2db.mulan.mulanarff with parameters of type DbManagers

    Constructor and Description

    `IdMultiLabelInstances(int arffId,
    DbManagers dbMan)`

    `MulanArff(java.lang.String dataQueriesProps,
    DbManagers dbMan)`

    `MulanArff(java.lang.String dataQueriesProps,
    DbManagers dbMan,
    boolean massive)`
  - ### Uses of DbManagers in main.ml2db.mulan.mulanarff.database

    Methods in main.ml2db.mulan.mulanarff.database that return DbManagers

    | Modifier and Type | Method and Description |
    | --- | --- |
    |  |  |
    | --- | --- |
    | `DbManagers` | MulanArffRecord.`getDbManagers()` |

    Methods in main.ml2db.mulan.mulanarff.database with parameters of type DbManagers

    | Modifier and Type | Method and Description |
    | --- | --- |
    |  |  |
    | --- | --- |
    | `static mulan.data.MultiLabelInstances` | MulanArffInstancesLoader.`generateDataSet(int arffRecordId, DbManagers dbMan)` |
    | `static mulan.data.MultiLabelInstances` | MulanArffInstancesLoader.`generateDataSet(java.lang.String arffFilePath, java.lang.String xmlFilePath, DbManagers dbMan)` |
    | `static IdMultiLabelInstances` | MulanArffInstancesLoader.`generateIdInstances(int arffRecordId, DbManagers dbMan)` |

    Constructors in main.ml2db.mulan.mulanarff.database with parameters of type DbManagers

    Constructor and Description

    `MulanArffRecord(int arffRecordId,
    DbManagers dbMan)`
  - ### Uses of DbManagers in main.ml2db.weka

    Methods in main.ml2db.weka that return DbManagers

    | Modifier and Type | Method and Description |
    | --- | --- |
    |  |  |
    | --- | --- |
    | `DbManagers` | Arff.`getDbManagers()` |

    Methods in main.ml2db.weka with parameters of type DbManagers

    | Modifier and Type | Method and Description |
    | --- | --- |
    |  |  |
    | --- | --- |
    | `void` | Arff.`checkAndInitialise(java.lang.String dataPropsFilePath, DbManagers dbMan, boolean massive)` |
    | `int` | ArffRecordsTable.`getIdByArffPath(java.lang.String arffPath, java.lang.String arffPropsFilePath, DbManagers dbMan)` Returns the first arff id in the table whose arff path matches the given string (or part of it). |
    | `static int` | Arff.`run(java.lang.String arffPropsPath, DbManagers dbMan, boolean massive)` |

    Constructors in main.ml2db.weka with parameters of type DbManagers

    Constructor and Description

    `Arff(java.lang.String dataPropsFilePath,
    DbManagers dbMan)`

    `Arff(java.lang.String dataPropsFilePath,
    DbManagers dbMan,
    boolean massive)`
  - ### Uses of DbManagers in test.ml2db

    Methods in test.ml2db that return DbManagers

    | Modifier and Type | Method and Description |
    | --- | --- |
    |  |  |
    | --- | --- |
    | `static DbManagers` | InputAndOutputDbManagersTest.`getInputAndOutputDbManagersDbManager()` |
  - ### Uses of DbManagers in test.ml2db.alltests

    Methods in test.ml2db.alltests that return DbManagers

    | Modifier and Type | Method and Description |
    | --- | --- |
    |  |  |
    | --- | --- |
    | `static DbManagers` | DbManagerForTest.`getTestDbManagers()` |
  - ### Uses of DbManagers in test.ml2db.dataharness

    Methods in test.ml2db.dataharness that return DbManagers

    | Modifier and Type | Method and Description |
    | --- | --- |
    |  |  |
    | --- | --- |
    | `static DbManagers` | TestProjectParameters.`getArffRealRestTestDbManagers()` |
    | `static DbManagers` | TestProjectParameters.`getDbManagers()` |
  - ### Uses of DbManagers in test.ml2db.weka

    Methods in test.ml2db.weka that return DbManagers

    | Modifier and Type | Method and Description |
    | --- | --- |
    |  |  |
    | --- | --- |
    | `static DbManagers` | ArffPropsTableTest.`getDbManagers()` |

- Overview
- Package
- Class
- Use
- Tree
- Deprecated
- Index
- Help

- Prev
- Next

- Frames
- No Frames

- All Classes
